# Individualized transcranial focused ultrasound stimulation improves cognitive impairment in Alzheimer's disease patients by enhancing the default mode network

**DOI:** 10.1002/alz70857_098945

**Published:** 2025-12-24

**Authors:** Jianwei Ge, Yi Ling, Benyan Luo

**Affiliations:** ^1^ First Affiliated Hospital, School of Medicine, Zhejiang University, Hangzhou, Zhejiang, China; ^2^ Department of Neurology, First Affiliated Hospital, School of Medicine, Zhejiang University, Hangzhou, Zhejiang, China; ^3^ First Affiliated Hospital, Zhejiang University, Hangzhou, Zhejiang, China

## Abstract

**Background:**

Alzheimer's disease (AD) is a progressive neurodegenerative disorder characterized by cognitive decline, memory loss, and behavioral changes. Current treatment options are limited in their ability to halt or reverse disease progression, leading to a growing interest in non‐invasive neuromodulation techniques. Transcranial focused ultrasound (tFUS) has emerged as a promising therapeutic approach due to its ability to modulate neural activity, enhance drug delivery across the blood‐brain barrier, and promote neuroplasticity. Recent studies suggest that tFUS may help alleviate AD symptoms by targeting specific brain regions involved in cognitive function, offering a potential avenue for non‐pharmacological intervention.

**Method:**

Using tFUS to treat AD patients continuously for two weeks. Neuropsychological assessments, resting‐state electroencephalography (EEG), resting‐state functional magnetic resonance imaging (fMRI), graph theory analysis, and edge analysis will be used to evaluate changes in cognitive function, brain network connectivity, EEG power spectral density, and functional connectivity before and after tFUS treatment in AD patients.

**Result:**

After two weeks of tFUS treatment for Alzheimer's disease patients, significant improvements were observed in MMSE and MOCA scores in the active stimulation group. Edge analysis revealed a significant enhancement in intra‐network connectivity within the default mode network (DMN) in the active stimulation group. DTI results showed a significant increase in the rich‐club coefficient in the active stimulation group. Resting‐state EEG indicated a significant reduction in delta band power spectral density in the frontal region of the scalp.

**Conclusion:**

TFUS stimulation has shown promising potential in improving cognitive impairment in Alzheimer's disease patients by enhancing the connectivity and functionality of the DMN. Additionally, neuroimaging findings have demonstrated that tFUS enhances structural and functional connectivity in key DMN regions, potentially slowing disease progression and offering a non‐invasive therapeutic strategy for managing cognitive decline in AD patients.

